# Stage IA papillary and chromophobe renal cell carcinoma: effectiveness of cryoablation and partial nephrectomy

**DOI:** 10.1186/s13244-024-01749-x

**Published:** 2024-07-06

**Authors:** Annemarie Uhlig, Johannes Uhlig, Brian Shuch, Hyun S. Kim

**Affiliations:** 1 Department of Urology, University Medical Center Goettingen, Goettingen, Germany; 2 Department of Diagnostic and Interventional Radiology, University Medical Center Goettingen, Goettingen, Germany; 3 Division of Vascular and Interventional Radiology, Department of Diagnostic Radiology and Nuclear Medicine, University of Maryland Marlene and Stewart Greenebaum Comprehensive Cancer Center, University of Maryland School of Medicine, Baltimore, MD USA; 4 Institute of Urologic Oncology, David Geffen School of Medicine at UCLA, Los Angeles, CA USA

**Keywords:** Cryoablation, Thermal ablation, Partial nephrectomy, Papillary renal cell carcinoma, Chromophobe renal cell carcinoma

## Abstract

**Objectives:**

To evaluate the effectiveness of cryoablation compared to partial nephrectomy in patients with stage IA papillary and chromophobe renal cell carcinoma (pRCC; chRCC).

**Material and methods:**

The 2004–2016 National Cancer Database was queried for adult patients with stage IA pRCC or chRCC treated with cryoablation or partial nephrectomy. Patients receiving systemic therapy or radiotherapy, as well as those with bilateral RCC or prior malignant disease were excluded. Overall survival (OS) was assessed using Kaplan–Meier plots and Cox proportional hazard regression models. Nearest neighbor propensity matching (1:1 cryoablation:partial nephrectomy, stratified for pRCC and chRCC) was used to account for potential confounders.

**Results:**

A total of 11122 stage IA renal cell carcinoma patients were included (pRCC 8030; chRCC 3092). Cryoablation was performed in 607 (5.5%) patients, and partial nephrectomy in 10515 (94.5%) patients. A higher likelihood of cryoablation treatment was observed in older patients with non-private healthcare insurance, as well as in those with smaller diameter low-grade pRCC treated at non-academic centers in specific US geographic regions. After propensity score matching to account for confounders, there was no statistically significant difference in OS comparing cryoablation vs partial nephrectomy in patients with pRCC (HR = 1.3, 95% CI: 0.96–1.75, *p* = 0.09) and those with chRCC (HR = 1.38, 95% CI: 0.67–2.82, *p* = 0.38).

**Conclusion:**

After accounting for confounders, cryoablation, and partial nephrectomy demonstrated comparable OS in patients with stage IA papillary and chromophobe RCC. Cryoablation is a reasonable treatment alternative to partial nephrectomy for these histological RCC subtypes when radiologically suspected or diagnosed after biopsy.

**Critical relevance statement:**

Cryoablation might be considered as an upfront treatment alternative to partial nephrectomy in patients with papillary and chromophobe stage IA renal cell carcinoma, as both treatment approaches yield comparable oncological outcomes.

**Key Points:**

- The utilization of cryoablation for stage IA papillary and chromophobe RCC increases.
- In the National Cancer Database, we found specific patterns of use of cryoablation.
- Cryoablation and partial nephrectomy demonstrate comparable outcomes after accounting for confounders.

**Graphical Abstract:**

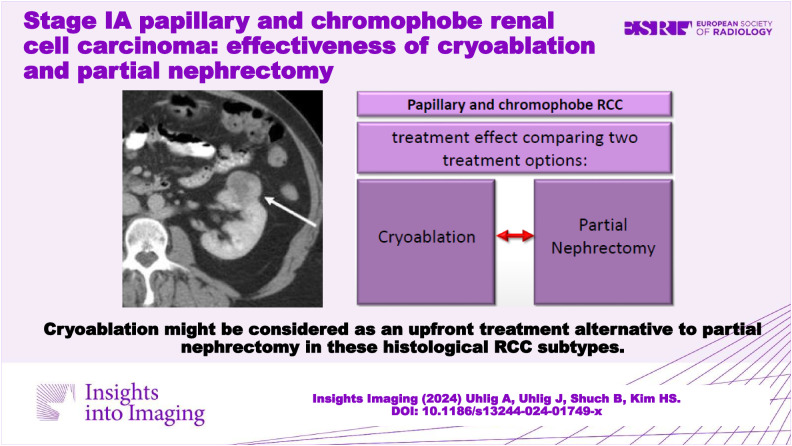

## Introduction

Renal cancer is the most common malignancy of the kidneys, accounting for a total of 431,288 cancer cases and 179,368 cancer deaths worldwide in 2020 [1]. Over the last decades, there has been an increase in the incidence of renal cancer cases. This has been partially attributed to technical advancements and a wider utilization of radiological cross-sectional imaging with localized tumors being detected at a smaller diameter [2]. Often, these tumors are localized to the kidney with a diameter of ≤ 4 cm (T1a) or ≤ 7 cm (T1b), and without nodal or distant metastases (AJCC stage I A/B according to T stage) [3].

Approximately 90–95% of renal cancers are renal cortical tumors termed “renal cell carcinomas” (RCC), with the most frequent histological subtypes being clear cell, papillary, and chromophobe RCCs (ccRCC, pRCC, and chRCC) accounting for > 90% of cases [4]. These histological subtypes have distinct cellular origins and prognostic profiles, with improved overall survival (OS) for patients with pRCC and chRCC [5, 6].

Surgical resection with partial nephrectomy (PN) has remained the mainstay of treatment for early-stage RCC [7, 8]. However, thermal ablation is an option for smaller diameter T1a tumors, as well as an alternative for selected patients with T1b tumors, i.e., if frail and/or comorbid [7–9]. Cryoablation (CRA) is an ablation technique that utilizes freezing and thaw cycles to induce tumoral cell death by disruption of cellular membranes by ice crystals and ischemic injury [10]. For RCC treatment, high technical CRA success rates of more than 95% have been reported [11]. Only few CRA patients suffer procedural complications and renal function decline, with the most frequent complications being low-grade, such as flank pain in up to 7% of patients [12]. Still, local recurrence is observed in up to 7.7% of T1a RCCs treated with CRA and increases with larger tumor diameter [13, 14].

In recent years, the pre-treatment identification of renal carcinoma subtypes has improved considerably, i.e., using CT-based radiomics or PET-CT [15, 16]. Knowledge of the renal tumor subtype before definitive treatment can then impact clinical decision-making with subtype-tailored treatment strategies.

So far, the majority of studies comparing PN to CRA have focused on the most common RCC clear-cell subtype, with a lack of data on the effectiveness of CRA in less common RCC subtypes [17]. In particular, given the distinct cellular origins and prognoses of ccRCC compared to pRCC and chRCC, further analyses are needed to guide clinical decision-making and individualized treatment plans. This study therefore evaluated the treatment effectiveness of CRA compared to PN for patients with pRCC and chRCC. We hypothesize that treatment outcomes of CRA and PN are comparable irrespective of the histological subtype.

## Methods

This study is Health Insurance Portability and Accountability Act (HIPAA) compliant and was approved by the institutional review board at Yale University.

### Patient population

Patients were included in the National Cancer Database (NCDB), which is jointly sponsored by the American College of Surgeons and the American Cancer Society. The NCDB contains approximately 34 million records from hospital cancer registries in the United States of America and captures approximately 70% of newly diagnosed annual cancer cases [18].

The 2019 version of the NCDB (reporting on patients diagnosed between 2004 and 2016) was searched for cases of stage IA renal cell cancer (RCC) with a tumor diameter of ≤ 40 mm. Inclusion criteria were histopathologic diagnosis confirmation of chromophobe or papillary histology, age 18 years and older, and treatment with CRA (surgical code 13) or partial nephrectomy (PN; surgical code 30). Exclusion criteria were systemic therapy, radiation, and non-primary RCC diagnosis (i.e., RCC diagnosis after lymphoma) except non-melanoma skin cancer, which was not reported in the NCDB. These exclusion criteria were selected to avoid confounding of survival outcomes by secondary malignancies.

### Variables

Histological RCC subtypes were stratified as papillary RCC (ICD-03 codes: 8050, 8260), and chromophobe RCC (ICD-03 code: 8317, 8270). Comorbidities at the time of RCC diagnosis were measured using the Charlson comorbidity index (CCI), stratified into CCI 0, CCI 1, CCI 2, and CCI ≥ 3. Geographic information was provided as broader US state regions. For patients < 39 years old, facility type and location were suppressed by the NCDB, as individual small strata of young patients at specific institution types and geographic locations might have jeopardized anonymization. Further variables included patient age, gender, race, insurance status, household income, educational status, histological cancer grade, RCC diameter, and year of diagnosis.

### Outcome

The primary outcome was OS, which was defined as the time from RCC diagnosis until death or censoring, whatever occurred first. The secondary outcome was the evaluation of demographic differences among treatment arms in papillary and chromophobe RCC patients.

### Matching

A nearest-neighbor propensity score matching procedure was used to balance potential confounders between CRA and PN. Given the distribution of treatments, a ratio of 1:5 CRA:PN was chosen, where each CRA patient was matched with five suitable PN patients. Propensity score matching was performed in a stratified manner for both pRCC and chRCC, where CRA and PN patients were separately matched for each histological RCC subtype. The caliper width to identify matches for CRA and PN patients was set at 0.1 standard deviations of the propensity score, providing a strict matching process. The calculation of the propensity score for each RCC patient was based on a multivariable logistic regression model predicting RCC treatment with CRA. Demographic and tumor variables were considered as predictors for CRA treatment for the logistic regression model (further details see results section).

The Kolmogorov–Smirnov test with 500 bootstrap samples was used to assess the balancing of confounders in the matched cohort, with a null hypothesis of no difference in confounder distribution [19].

### Statistical analyses

For descriptive analyses, continuous variables were provided as median and inter-quartile range (IQR). Categorical variables were presented as absolute numbers and frequency. For the propensity score model, logistic regression was used to predict the probability of RCC treatment with CRA, considering variables with univariate *p* < 0.1 for inclusion in the multivariable model, and retaining them at multivariable *p* < 0.05. OS was assessed with OS rates and univariate Cox proportional hazards models. OS differences in subgroups with crossing Kaplan–Meier curves were calculated using supremum family tests with Breslow–Gehan weights. Median follow-up time was estimated using the reverse Kaplan–Meier method. All statistical analyses were performed with R version 3.4.3 (R Core Development Team, Vienna, Austria) and RStudio version 1.3.959 (RStudio Inc., Boston, MA). An alpha level of 0.05 was chosen for statistical significance. All *p*-values are two-sided.

## Results

### Patient population

**Fig. 1:**
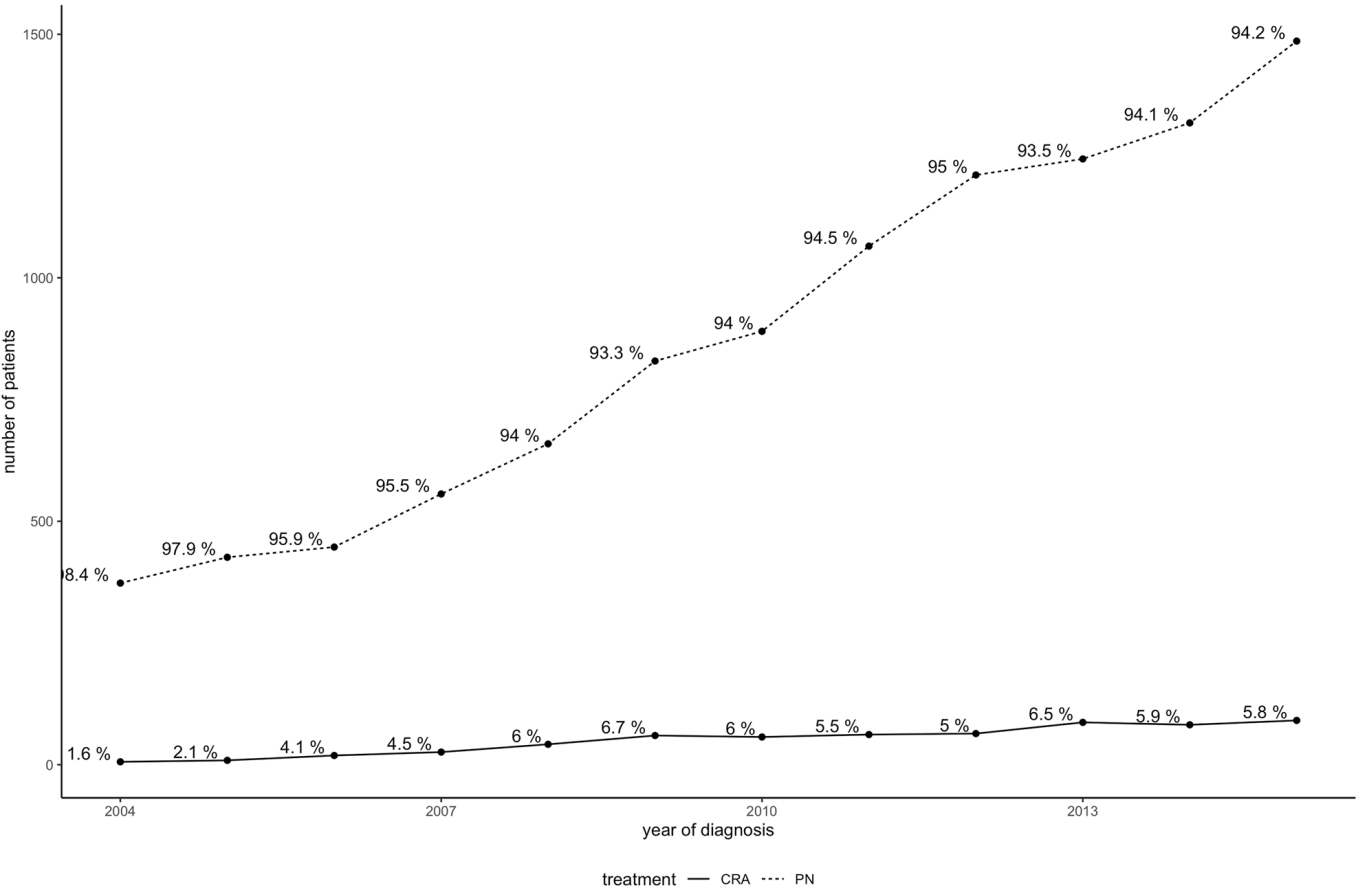
Temporal changes in RCC treatment over the study period

**Fig. 2:**
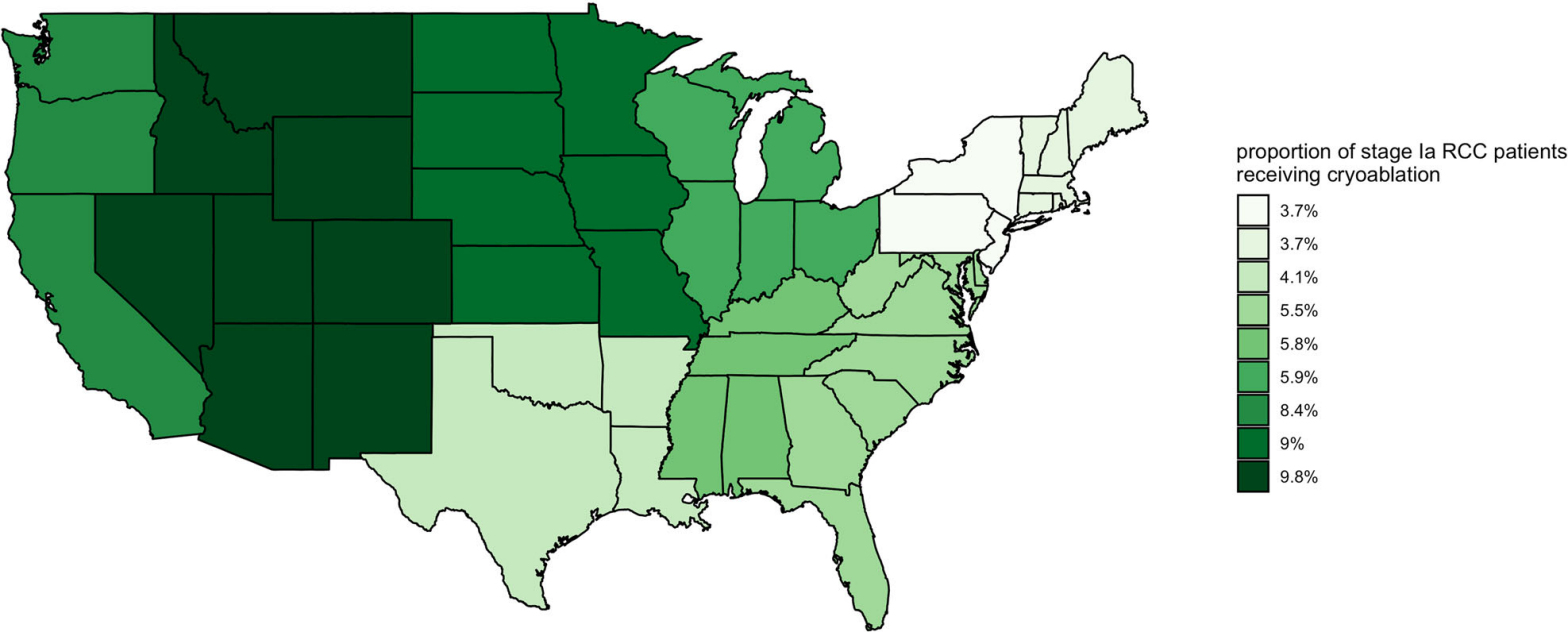
US geographical differences in CRA utilization for stage IA pRCC and chRCC

A total of 11,122 stage I RCC patients were included (pRCC 8030; chRCC 3092), of which 607 (5.5%) received CRA and 10,515 (94.5%) received PN. A study flowchart is provided in Appendix Fig. 1 (electronic supplementary material). Tables 1 and 2 show baseline characteristics of included patients. On multivariable logistic regression, a higher likelihood of CRA treatment was independently observed for older patients with non-private healthcare insurance (Appendix Table 1). CRA was more likely in patients with smaller diameter, low-grade pRCC, and at non-academic centers. Further, there was an independent time-trend with a higher likelihood of PN treatment in the later years of the study period (Table 1 and Fig. 1). As shown in Table 1 and Fig. 2, geographic discrepancies in RCC treatment were observed, with the highest CRA rates in the Mountain states (9.76%) and lowest in Middle Atlantic states (3.66%).

**Table 1:** Demographic characteristics of included patients

Parameter		Total	PN	CRA	*p*
*n*		11,122	10,515	607	
Age					< 0.01
	Median (IQR)	60 (52–68)	60 (52–67)	67 (59–75)	
Gender					0.06
	Female	3711 (33.4%)	3530 (33.6%)	181 (29.8%)	
	Male	7411 (66.6%)	6985 (66.4%)	426 (70.2%)	
Race					*0.03*
	White	8626 (77.6%)	8131 (77.3%)	495 (81.5%)	
	African American	2065 (18.6%)	1968 (18.7%)	97 (16.0%)	
	Others	431 (3.9%)	416 (4.0%)	15 (2.5%)	
Insurance					< 0.01
	Private insurance	5991 (53.9%)	5802 (55.2%)	189 (31.1%)	
	Medicare	4008 (36.0%)	3645 (34.7%)	363 (59.8%)	
	Medicaid	619 (5.6%)	588 (5.6%)	31 (5.1%)	
	Govt. insurance	152 (1.4%)	141 (1.3%)	11 (1.8%)	
	Not insured/unknown insurance	352 (3.2%)	339 (3.2%)	13 (2.1%)	
Annual household income in the residency area					< 0.01
	< $40,227	1945 (17.8%)	1852 (17.9%)	93 (15.5%)	
	$40,227–50,353	2224 (20.3%)	2078 (20.1%)	146 (24.3%)	
	$50,354–63,332	2386 (21.8%)	2226 (21.5%)	160 (26.7%)	
	≥ $63,333	4391 (40.1%)	4190 (40.5%)	201 (33.5%)	
Proportion of residents without high school diploma in residency area					0.09
	≥ 17.6%	2096 (19.1%)	2002 (19.3%)	94 (15.6%)	
	10.9–17.5%	2777 (25.3%)	2615 (25.2%)	162 (27.0%)	
	6.3–10.8%	3081 (28.1%)	2896 (27.9%)	185 (30.8%)	
	< 6.3%	3012 (27.5%)	2852 (27.5%)	160 (26.6%)	
Comorbidities (Charlson Deyo comorbidity index)					0.01
	0	8092 (72.8%)	7676 (73.0%)	416 (68.5%)	
	1	2318 (20.8%)	2184 (20.8%)	134 (22.1%)	
	2	522 (4.7%)	481 (4.6%)	41 (6.8%)	
	≥ 3	190 (1.7%)	174 (1.7%)	16 (2.6%)

**Table 2:** Tumor and treatment-related parameters of included patients

Parameter		Total	PN	CRA	*p*
Cancer grade					< 0.01
	Grade I	1205 (10.8%)	1119 (10.6%)	86 (14.2%)	
	Grade II	4708 (42.3%)	4553 (43.3%)	155 (25.5%)	
	Grade III	1967 (17.7%)	1944 (18.5%)	23 (3.8%)	
	Grade IV	143 (1.3%)	143 (1.4%)	0 (0.0%)	
	Grade unknown	3099 (27.9%)	2756 (26.2%)	343 (56.5%)	
Cancer diameter					< 0.01
	1 cm and less	348 (3.1%)	339 (3.2%)	9 (1.5%)	
	1.1–2 cm	3396 (30.5%)	3171 (30.2%)	225 (37.1%)	
	2.1–3 cm	4467 (40.2%)	4204 (40.0%)	263 (43.3%)	
	3.1–4 cm	2911 (26.2%)	2801 (26.6%)	110 (18.1%)	
Histology					< 0.01
	Chromophobe RCC	3092 (27.8%)	2974 (28.3%)	118 (19.4%)	
	Papillary RCC	8030 (72.2%)	7541 (71.7%)	489 (80.6%)	
Year of cancer diagnosis					< 0.01
	Median (IQR)	2012 (2009–2014)	2012 (2009–2014)	2012 (2009–2014)	
Facility type					< 0.01
	Academic/research center	5393 (48.5%)	5140 (48.9%)	253 (41.7%)	
	Non-academic center	5729 (51.5%)	5375 (51.1%)	354 (58.3%)	
Facility location					< 0.01
	East North Central	1952 (17.6%)	1837 (17.5%)	115 (18.9%)	
	East South Central	737 (6.6%)	694 (6.6%)	43 (7.1%)	
	Facility location suppressed for age 0–39 years	616 (5.5%)	607 (5.8%)	9 (1.5%)	
	Middle Atlantic	2184 (19.6%)	2104 (20.0%)	80 (13.2%)	
	Mountain	379 (3.4%)	342 (3.3%)	37 (6.1%)	
	New England	669 (6.0%)	644 (6.1%)	25 (4.1%)	
	Pacific	943 (8.5%)	864 (8.2%)	79 (13.0%)	
	South Atlantic	2096 (18.8%)	1980 (18.8%)	116 (19.1%)	
	West North Central	809 (7.3%)	736 (7.0%)	73 (12.0%)	
	West South Central	737 (6.6%)	707 (6.7%)	30 (4.9%)

### CRA effectiveness in patients with papillary RCC

**Fig. 3:**
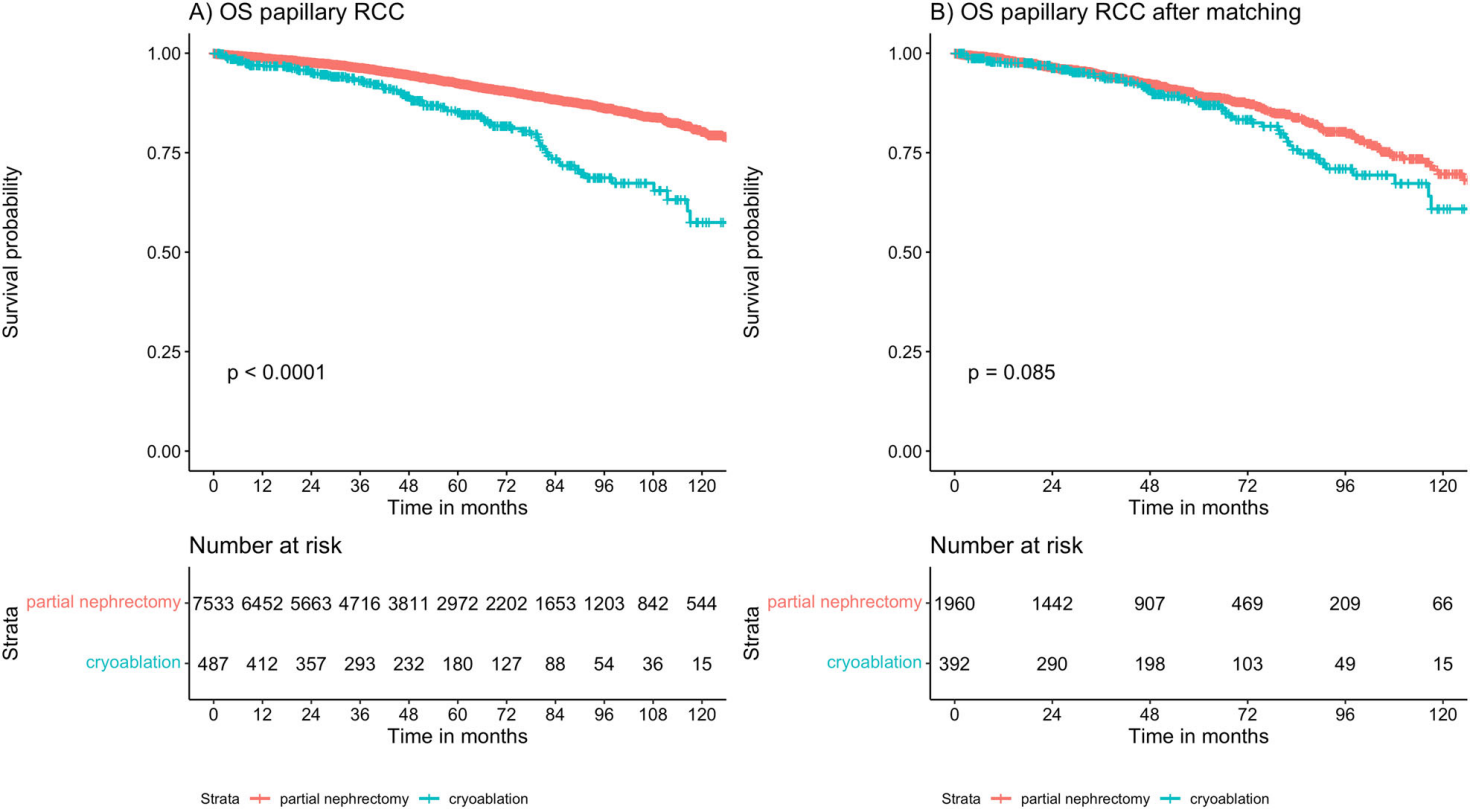
OS depending on treatment with CRA and PN in the unmatched (**A**) and matched (**B**) cohort of patients with pRCC

Among a total of 8030 patients with pRCC included in this study, 489 (6.1%) received CRA. Median follow-up time among patients with pRCC was 51.7 months (IQR: 25.6–83.8 months). After propensity score matching, a matched cohort of 2352 pRCC patients (CRA 392; PN 1960) with a balanced distribution of confounders was obtained (see Appendix Table 1). In the unmatched pRCC cohort, shorter OS was evident when comparing CRA to PN (hazard ratio, HR = 2.22, 95% confidence interval (CI):1.75–2.82, *p* < 0.001). However, in the matched pRCC cohort, comparable OS for CRA and PN was seen (HR = 1.3, 95% CI: 0.96–1.75, *p* = 0.09). OS depending on treatment in pRCC is depicted in Fig. 3.

### CRA effectiveness in patients with chromophobe RCC

**Fig. 4:**
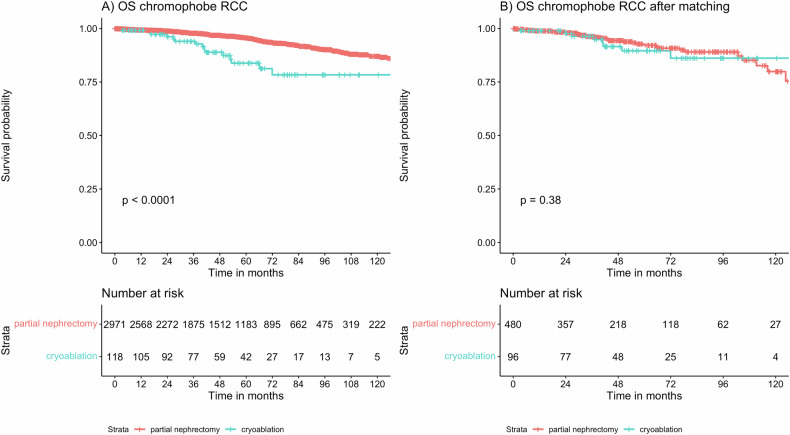
OS depending on treatment with CRA and PN in the unmatched (**A**) and matched (**B**) cohort of patients with chRCC

A total of 3092 patients with chRCC were included in this study, of which 118 (1.5%) received CRA for renal tumor ablation. The median follow-up time for chRCC was 50.6 months (IQR: 26.3–82.3 months). After propensity score matching, a matched cohort of 576 chRCC patients (CRA 96; PN 480) with a balanced distribution of confounders was obtained (Table 2). Among the unmatched chRCC patients, shorter OS was evident when comparing treatment with CRA to PN (HR = 3.33, 95% CI: 2.04–5.44, *p* < 0.001). However, after confounder adjustment using propensity score matching, comparable OS was seen for CRA and PN (HR = 1.38, 95% CI: 0.67–2.82, *p* = 0.38). OS depending on treatment in the chRCC cohort is depicted in Fig. 4.

## Discussion

Thermal ablation is an option for smaller diameter T1a renal tumors, as well as an alternative to PN in selected patients with T1b tumors, i.e., if frail and/or comorbid [7, 8]. However, to date, there is a lack of data on CRA effectiveness depending on the histological RCC subtype, with most renal ablation studies focusing on patients with clear cell RCC [17]. In light of recent advances in pre-treatment identification of renal tumor subtypes, the effectiveness of CRA must be evaluated for the most common RCC subtypes to provide individualized treatment plans [15, 16].

This study demonstrates that treatment effectiveness of CRA and PN is comparable in patients with stage IA pRCC and chRCC: after accounting for potential confounders, no statistically significant OS difference was evident for CRA and PN in patients with pRCC (HR = 1.3, 95% CI: 0.96–1.75, *p* = 0.09) and chRCC (HR = 1.38, 95% CI: 0.67–2.82, *p* = 0.38). To the best of our knowledge, this study is the first to evaluate CRA effectiveness in comparison to PN for pRCC and chRCC [17]. Comparable to our findings, the effectiveness of RCC thermal ablation using heat-based radiofrequency ablation varied according to histological subtype in a cohort of 229 cT1a RCCs: across two institutions, Lay et al demonstrated numerically shorter disease-free survival in patients with ccRCC vs papillary RCC with a 5-year disease-free survival of 89.7% vs 100% [20]. Similarly, Haddad et al reported on 43 pRCC patients treated with CRA from a single center and reported a 5-year disease-free survival rate of 100% [21].

Several mechanisms may underlie these findings. Although the literature is equivocal regarding the local control rates after CRA compared to PN, local tumor recurrence remains a concern for RCC ablation techniques [17, 22–27]. However, papillary and chromophobe RCC have been shown to be associated with a lower risk of local recurrence after surgical resection when compared to clear cell RCC, with recurrence-free-survival HR = 0.72 for chRCC and HR = 0.3 for pRCC vs ccRCC (both after multivariable adjustment for confounders) [28]. Other studies corroborated a lower risk of cancer-specific death or metastases for papillary and chromophobe vs clear cell RCC, i.e., demonstrating 5-year freedom from metastasis or death from disease of 86% in ccRCC, 95% in pRCC, and 92% in chRCC, respectively [29, 30]. Improved local control rates might therefore contribute to comparable OS of CRA and PN for papillary and chromophobe RCC, while also explaining the OS differences observed among patients with clear cell RCC. Since the NCDB does not provide details on disease recurrence, disease-free survival analyses were not possible. Still, OS evaluated in this study is probably the most relevant outcome impacting clinical decision-making. In particular, given the favorable complication profile of CRA, patients with chRCC and pRCC might opt for CRA if associated with OS that is comparable to PN.

Moreover, sporadic ccRCC is associated with an inactivation of the Von Hippel–Lindau gene in approximately 80% of cases, which leads to high tumor vascularization [31]. In contrast, these mutations are rarely observed in papillary and chromophobe RCC. Therefore, pRCC and chRCC could be less affected by the so-called “cold-sink” for CRA, postulated for tumors in proximity to vessels in the kidney, which could impact oncological outcomes [32, 33]. Further, Kluger et al have shown lower microvessel density in pRCC and chRCC compared to ccRCC, which could further affect the effectiveness of CRA in these specific subtypes [34].

The distribution of histological RCC subtypes reported in our study with a majority of pRCC vs. chRCC compares well to the so-far published literature [28]. Further, similar to the results in this study, a higher proportion of PN compared to thermal ablation, as well as geographical differences in the utilization of thermal ablation for RCC treatment across the United States have been reported earlier [35].

This study is not devoid of limitations, which are mainly inherent to its retrospective design evaluating the NCDB. First, only OS was available as an oncological outcome measure. Since the NCDB does not provide tumor recurrence or cause of death, analyses of recurrence-free and cancer-specific survival need to be conducted on other datasets. Second, no technical details were available concerning the CRA procedure, such as the number of probes and freeze-thaw cycles, as well as any procedural complications. Third, the exact location of the RCC within the kidney (e.g., as R.E.N.A.L. score) and renal function were not reported in the NCDB, which may have resulted in residual confounding. Further, given the low prevalence of the chromophobe RCC subtype and low utilization of CRA, subgroup analyses for chromophobe RCC might be statistically underpowered to detect a true difference in OS. Finally, the NCDB did not provide details on subgroupings of papillary RCC as type I and type II with distinct prognostic outcomes, since no separate ICD-O3 codes are available.

## Conclusions

This study demonstrates that CRA and PN yield comparable OS in patients with stage IA pRCC and chRCC. These results might aid healthcare professionals in specifically tailoring RCC treatment plans in the era where pre-treatment histologic identification is more reliable. Still, further studies are needed to confirm these findings in a prospective, controlled manner while also evaluating additional oncological outcomes such as recurrence-free and cancer-specific survival.

### Supplementary information

ELECTRONIC SUPPLEMENTARY MATERIAL

## Data Availability

All patient data can be received from the NCDB.

## Abbreviations

CCI: Charlson comorbidity index
ccRCC: Clear cell renal cell carcinoma
chRCC: Chromophobe renal cell carcinoma
CI: Confidence interval
CRA: Cryoablation
HIPAA: Health Insurance Portability and Accountability Act
HR: Hazard ratio
IQR: Inter-quartile range
NCDB: National Cancer Database
OS: Overall survival
PN: Partial nephrectomy
pRCC: Papillary renal cell carcinoma
RCC: Renal cell carcinoma
