# Supplementary material for: Stage IA papillary and chromophobe renal cell carcinoma: effectiveness of cryoablation and partial nephrectomy
Source: Insights Imaging. 2024 Jul 6;15:171. doi: 10.1186/s13244-024-01749-x (PMC11227485; doi:10.1186/s13244-024-01749-x)
Supplement: Supplementary file 1 — ELECTRONIC SUPPLEMENTARY MATERIAL [file 13244_2024_1749_MOESM1_ESM.pdf]

Stage IA Papillary and Chromophobe Renal Cell Carcinoma: Effectiveness of Cryoablation and Partial Nephrectomy
ELECTRONIC SUPPLEMENTARY MATERIAL

Appendix figure 1: Study flowchart

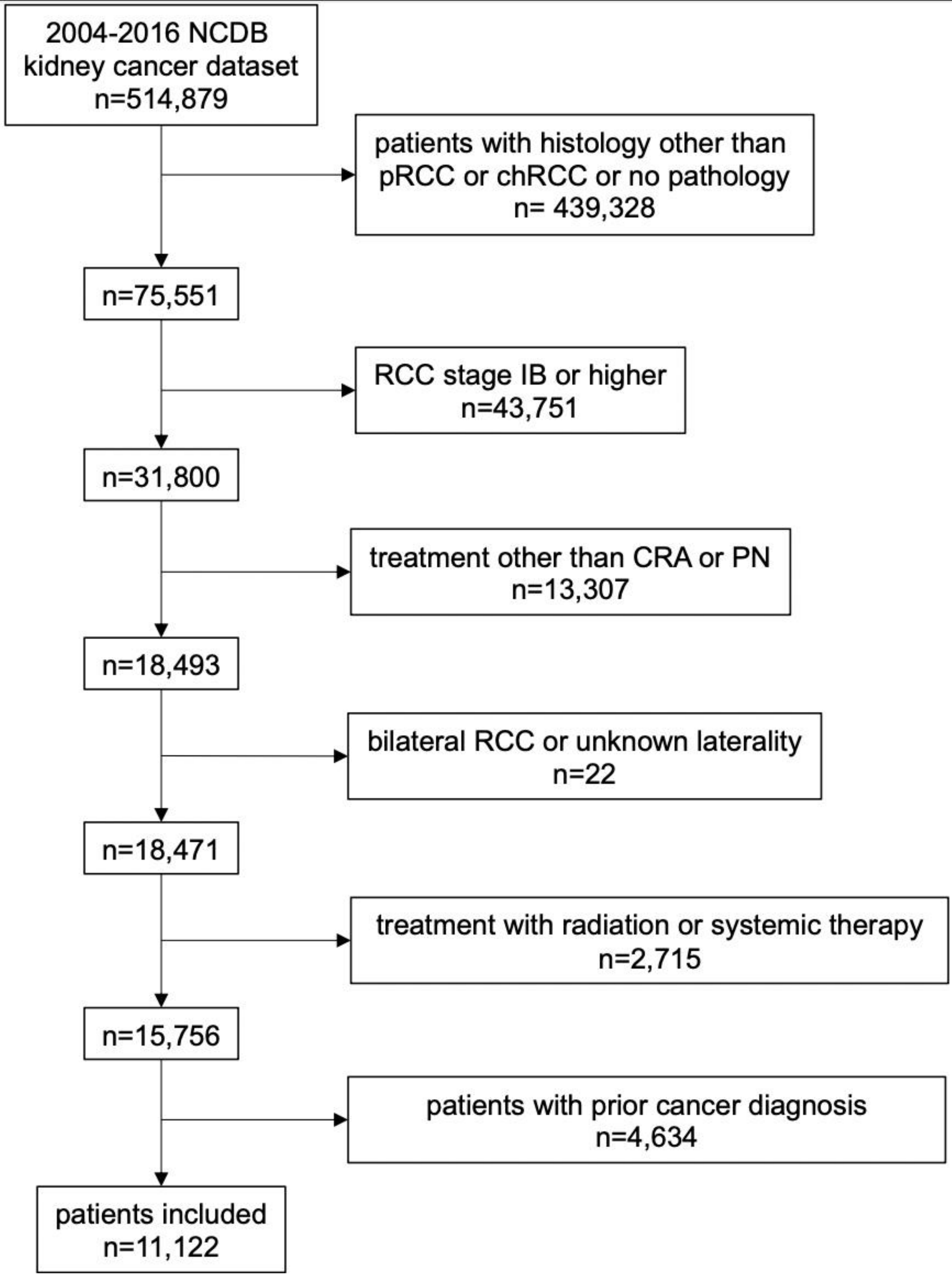

Appendix table 1: Univariate and multivariable logistic regression model papillary and chromophobe stage IA treatment with CRA.

variable	levels	univariate OR (95% CI)	multivariable OR (95% CI)
age	per 1 year increment	1.06 (1.06-1.07, p<0.001)	1.06 (1.04-1.07, p<0.001)
gender	female	1 (reference)	-
	male	1.19 (1.00-1.42, p=0.057)	-
race	white	1 (reference)	-
	african american	0.81 (0.64-1.01, p=0.064)	-
	others	0.59 (0.34-0.96, p=0.050)	-
insurance	private insurance	1 (reference)	-
	Medicare	3.06 (2.56-3.67, p<0.001)	1.47 (1.16-1.86, p=0.001)
	Medicaid	1.62 (1.08-2.35, p=0.015)	1.80 (1.18-2.66, p=0.005)
	govt. insurance	2.39 (1.20-4.30, p=0.007)	1.52 (0.75-2.83, p=0.211)
	not insured/unknown insurance	1.18 (0.63-2.01, p=0.576)	1.08 (0.57-1.89, p=0.795)
annual household income in residency area	< $40,227	1 (reference)	-
	$40,227-50,353	1.40 (1.07-1.83, p=0.014)	-
	$50,354-63,332	1.43 (1.10-1.87, p=0.007)	-
	>=$63,333	0.96 (0.75-1.23, p=0.722)	-
	(Missing)	-	-
proportion of residents without highschool diploma in residency area	>=17.6%	1 (reference)	-
	10.9-17.5%	1.32 (1.02-1.72, p=0.037)	-
	6.3-10.8%	1.36 (1.06-1.76, p=0.018)	-
	<6.3%	1.19 (0.92-1.56, p=0.181)	-
	(Missing)	-	-
comorbidities (Charlson Deyo Comorbidity Index)	0	1 (reference)	-
	1	1.13 (0.92-1.38, p=0.225)	-

	2	1.57 (1.11-2.17, p=0.008)	-
	>=3	1.70 (0.97-2.77, p=0.047)	-
histology	chromophobe RCC	1 (reference)	1 (reference)
	papillary RCC	1.63 (1.34-2.02, p<0.001)	2.19 (1.76-2.74, p<0.001)
cancer grade	Grade I	1 (reference)	1 (reference)
	Grade II	0.44 (0.34-0.58, p<0.001)	0.44 (0.33-0.59, p<0.001)
	Grade III	0.15 (0.09-0.24, p<0.001)	0.15 (0.09-0.23, p<0.001)
	Grade IV	0.00 (0.00-0.00, p=0.944)	0.00 (0.00-0.00, p=0.943)
	Grade unknown	1.62 (1.27-2.08, p<0.001)	2.02 (1.56-2.64, p<0.001)
cancer diameter	1.1 - 2cm	1 (reference)	1 (reference)
	1cm and less	0.37 (0.18-0.69, p=0.004)	0.30 (0.14-0.57, p=0.001)
	2.1 - 3cm	0.88 (0.73-1.06, p=0.179)	0.84 (0.69-1.02, p=0.077)
	3.1 - 4cm	0.55 (0.44-0.70, p<0.001)	0.48 (0.37-0.61, p<0.001)
year of cancer diagnosis	per 1 year increment	1.05 (1.02-1.08, p<0.001)	1.03 (1.00-1.06, p=0.045)
facility type	academic/research center	1 (reference)	1 (reference)
	non-academic center	1.34 (1.13-1.58, p=0.001)	1.26 (1.05-1.51, p=0.014)
facility location	East North Central	1 (reference)	1 (reference)
	East South Central	0.99 (0.68-1.41, p=0.955)	1.11 (0.75-1.61, p=0.590)
	facility location suppressed for age 0-39 years	0.24 (0.11-0.44, p<0.001)	1.33 (0.58-2.72, p=0.468)
	Middle Atlantic	0.61 (0.45-0.81, p=0.001)	0.69 (0.51-0.94, p=0.021)
	Mountain	1.73 (1.16-2.52, p=0.006)	1.85 (1.20-2.78, p=0.004)
	New England	0.62 (0.39-0.95, p=0.034)	0.65 (0.41-1.01, p=0.067)
	Pacific	1.46 (1.08-1.96, p=0.013)	1.75 (1.27-2.40, p=0.001)
	South Atlantic	0.94 (0.72-1.22, p=0.625)	1.03 (0.78-1.36, p=0.854)
	West North Central	1.58 (1.16-2.15, p=0.003)	2.24 (1.61-3.10, p<0.001)
	West South Central	0.68 (0.44-1.01, p=0.064)	0.74 (0.48-1.12, p=0.171)
